# T.O.HO.-PCNL Score for Predicting Success of Percutaneous Nephrolithotripsy: A New Score Development Study

**DOI:** 10.3390/jcm15020409

**Published:** 2026-01-06

**Authors:** Şeref Coser, Okan Alkıs, Baki Numan Özkaynak, Samet Senel, Halil İbrahim İvelik, İbrahim Güven Kartal, Bekir Aras

**Affiliations:** 1 Department of Urology, School of Medicine, Kutahya University of Health Sciences, Kutahya 43100, Turkey; okanalks@hotmail.com (O.A.); drbakinumanozkaynak@gmail.com (B.N.Ö.); halib_ive@hotmail.com (H.İ.İ.); igk84@hotmail.com (İ.G.K.); bekiraras1@gmail.com (B.A.); 2 Department of Urology, Ankara City Hospital, Ankara 06800, Turkey; samet_senel_umt@hotmail.com

**Keywords:** T.O.HO.-PCNL score, predicting PCNL success

## Abstract

**Background/Objectives:** The aim of this study was to develop a modified version of the T.O.HO. score for predicting percutaneous nephrolithotomy (PCNL) success. **Materials and Methods:** Patient demographics, stone-related parameters, perioperative findings, and postoperative outcomes were recorded. We reviewed the data of 155 patients who underwent percutaneous nephrolithotomy (PCNL) between October 2020 and December 2024. Patients were divided into two groups: success and failure. While preserving the validated components of the existing T.O.HO. score, the stone location parameter was restructured to more accurately reflect the anatomic challenges inherent to PCNL and was scored to include locations in the renal pelvis, upper, middle, lower and multiple calyces. The performance of the T.O.HO.-PCNL score in predicting surgical success was evaluated using ROC curve analysis. **Results:** The overall success rate was 65.8%. Patients in the successful group had smaller stone sizes and shorter operative times and hospital stays (*p* < 0.01). Preoperative hydronephrosis was more commonly observed among unsuccessful group (*p* < 0.05). The T.O.HO.-PCNL score was significantly lower in the successful group compared with the unsuccessful group. (*p* < 0.05). In the multivariate logistic regression analysis, stone size emerged as an independent predictor of PCNL success (OR: 1.076; 95% CI: 1.032–1.122; *p* < 0.001). ROC curve analysis demonstrated that the T.O.HO.-PCNL score had predictive value for PCNL success, with an optimal cut-off of 8.5 (AUC: 0.598; 95% CI: 0.506–0.690; *p* = 0.046). **Conclusions:** The T.O.HO.-PCNL scoring system is a promising nomogram for predicting stone-free status after PCNL in preoperative evaluation.

## 1. Introduction

Nephrolithiasis is a significant urological disease with a worldwide increasing prevalence and high recurrence rates [1]. In the general population, the lifetime probability of developing urinary tract stones is approximately 13% in men, while this rate is approximately 7% in women [2]. In recent years, significant advances in surgical treatment methods for kidney stones have been made in parallel with advances in technology. According to current guidelines, percutaneous nephrolithotomy (PCNL) continues to be the preferred first-line option for kidney stones exceeding 2 cm [3]. Stone-free rates (SFRs), the main variable of PCNL success, are reported in the literature to range from 56% to 98% [4]. Although PCNL is pivotal kidney stone treatment, there is no gold standard method for estimating success and complication rates [5]. Stone- and patient-related factors are among the most important determinants of surgical success and directly influence the choice of surgical method. Therefore, various scoring systems have been created to evaluate these factors to determine the appropriate surgical method and increase success rates.

Thomas et al. proposed Guy’s Stone Score (GSS) in 2011, marking the development of the first nomogram for predicting PCNL success [6]. In addition, scoring systems such as Staghorn Morphometry, STONE Nephrolithometry (STONE), and the Clinical Research Office of the Society of Endourology (CROES) nomogram are also frequently used [7,8,9]. These scoring systems are intended to predict the probability of a successful result and the risk of complications in PCNL by evaluating variables such as stone size, stone location, stone number, and anatomical features. In 2020, Hori et al. proposed a robust scoring system based on Tallness (T), Occupied lesion (O), and Hounsfield unit (HO) parameters to predict the success of flexible ureterorenoscopy for kidney stones and named this system the T.O.HO. score [10]. To date, many external validation studies have been conducted for this score, and modified versions have also been developed [11,12,13]. In this study, we applied this score after modifying it for PCNL. Therefore, our study is not an external validation study in the classical sense; the purpose of this study was to create a revised T.O.HO. scoring model specifically applicable to PCNL.

## 2. Patients and Methods

### 2.1. Data Collection and Ethical Approval

**Figure 1:**
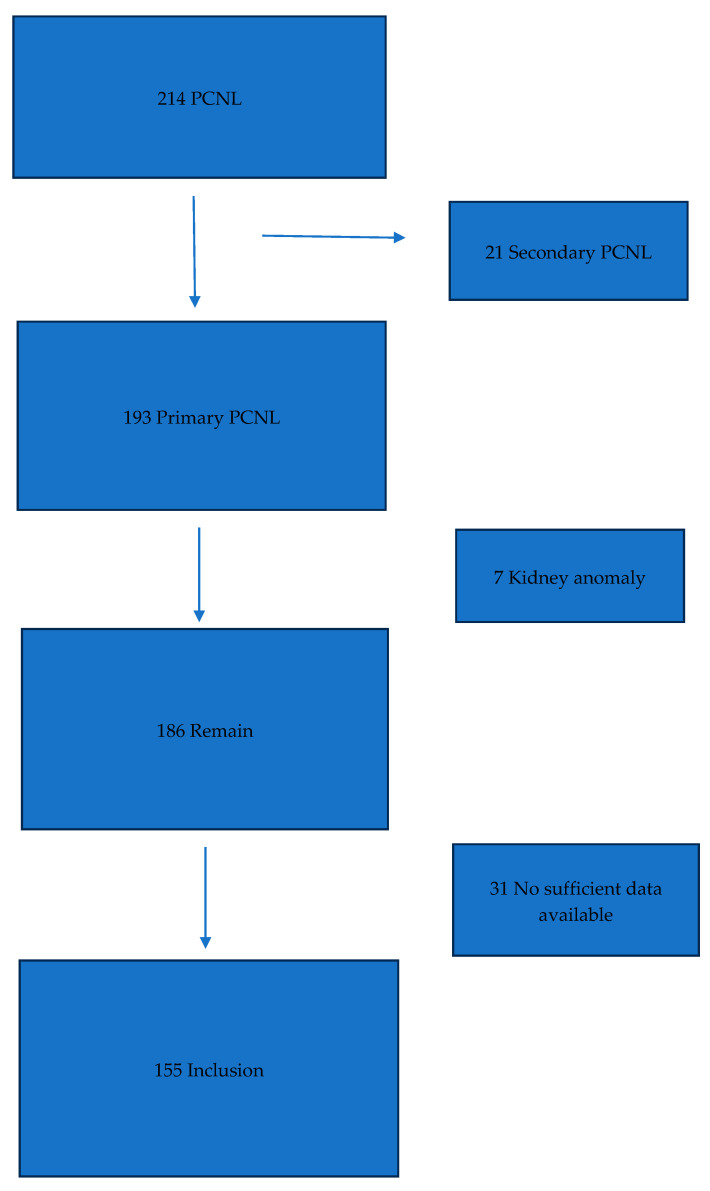
Flowchart of included patients.

After obtaining institutional approval from Ethics Committee of Kütahya Health Sciences University (Approval No: 2025/09-27) on the 27 September 2025, clinical data were obtained from patients who underwent percutaneous nephrolithotomy (PCNL) for renal calculi between October 2020 and December 2024. Among the 214 cases initially assessed, 21 with previous renal stone surgery history, 7 with structural renal anomalies, and 31 with incomplete clinical data were excluded. The final study cohort consisted of 155 patients (Figure 1).

### 2.2. Patient Characteristics and Preoperative Assessment

Patient-related data—such as age, sex, body mass index (BMI), duration of surgery, and hospitalization duration—were obtained from the medical records. All participants had undergone non-contrast computed tomography (NCCT) before preoperative period. Based on CT findings, stone laterality, number, maximal diameter (mm), localization, and density (Hounsfield Unit, HU) were recorded, along with the presence or absence of hydronephrosis.

Stone density was determined by averaging attenuation values measured at multiple regions (center, periphery, and mid-sections) of each calculus. For cases involving multiple stones, mean HU and cumulative diameters were calculated by averaging the individual values.

### 2.3. T.O.HO.-PCNL Score

The T.O.HO.-PCNL scoring system was designed to quantify procedural complexity using three radiologic parameters: stone size (T), location (O), and density (HO). Scores were assigned as follows:

- **Stone size (T):** <10 mm = 1, 10–17 mm = 2, 17–24 mm = 3, 24–30 mm = 4, >30 mm = 5
- **Stone location (O):** renal pelvis = 1, lower calyx = 2, middle calyx = 3, upper calyx = 4, multiple sites = 5
- **Stone density (HO):** <620 HU = 1, 620–1100 HU = 2, >1100 HU = 3

The overall score ranged between 3 and 13. Higher scores reflected greater procedural difficulty and lower anticipated success rates. Measurements were independently performed by two authors (Ş.C. and O.A.), with a third author (İ.G.K.) consulted when discrepancies arose.

### 2.4. PCNL Prosedure

All procedures were performed using a standardized prone PCNL approach. Fascial dilators of 8 Fr, 16 Fr, and 30 Fr were employed sequentially for tract dilation before introducing an Amplatz^®^ sheath (Boston Scientific Corporation, Marlborough, MA, USA)**.** A 24 Fr rigid nephroscope (Karl Storz, Munich, Germany) was used for nephroscopy, and lithotripsy was accomplished with a combined pneumatic-ultrasonic lithoclast (Swiss Lithoclast Master, Nyon, Switzerland). A 24 Fr nephrostomy tube was inserted at the completion of each operation. Postoperative evaluation included imaging of the kidneys, ureters, and bladder was performed on day 1 to assess residual fragments. All operations were conducted by four surgeons with a minimum of ten years’ endourological experience.

### 2.5. Clinical Outcomes

Patients were classified as “stone-free” if postoperative NCCT at 1 month revealed no residual calculi or only fragments ≤ 4 mm. Based on this criterion, cases were grouped as successful or unsuccessful. Between-group comparisons included T.O.HO.-PCNL scores, complication grades (Clavien–Dindo), need for additional interventions, and changes in hemoglobin and creatinine levels.

### 2.6. Statistical Analysis

Statistical analyses were conducted with IBM SPSS Statistics version 22.0 (IBM Corp., Chicago, IL, USA). The distribution of continuous variables was evaluated using the Shapiro–Wilk test. For data not conforming to a normal distribution, the Mann–Whitney U test was applied, while categorical variables were compared using either chi-square or Fisher’s exact tests as appropriate. Factors independently associated with PCNL outcomes were determined through multivariate logistic regression employing a backward likelihood ratio approach. The discriminatory performance of the T.O.HO.-PCNL scoring system was assessed via receiver operating characteristic (ROC) analysis with corresponding 95% confidence intervals. Statistical significance was defined as a two-tailed *p*-value below 0.05.

## 3. Results

A total of 155 patients were included in the study, with an overall stone-free success rate of 65.8%. There were no statistically significant differences between the successful and unsuccessful groups regarding age, gender, or BMI (*p* = 0.211, *p* = 0.265, and *p* = 0.051, respectively). Stone lateralization and anatomical location were also similar between the groups (*p* = 0.301 and *p* = 0.723). Although the mean stone density tended to be higher in the unsuccessful group, this difference did not reach statistical significance (1090.38 ± 347.12 HU vs. 990.75 ± 351.83 HU; *p* = 0.095). In contrast, the unsuccessful group had significantly larger stones (35.30 ± 11.50 mm vs. 27.45 ± 8.71 mm; *p* < 0.01). The presence of hydronephrosis was also more common among unsuccessful group (*p* < 0.05). Operative time and postoperative hospital stay were both significantly longer in the unsuccessful group (both *p* < 0.01). Additionally, T.O.HO.-PCNL score was significantly lower among successful patients compared to unsuccessful group (9.21 ± 2.27 vs. 9.96 ± 2.10; *p* < 0.05).

While the mean residual stone size was 0.51 ± 1.12 mm in the successful group, it was significantly higher in the unsuccessful group (5.96 ± 0.66 mm; *p* < 0.01). Additional treatments were used for residual stones in 30.2% of the patients in the unsuccessful group (16/53, ESWL for 7 patients, retrograde intrarenal surgery (RIRS) for 4 patients, URS for 3 patients, and a second session of PCNL for 2 patients). After additional interventions, 46 of 53 patients (86.8%) in this group achieved definitive stone-free status.

In the group achieving stone-free status, 61 patients had no recorded complications. A total of 25 patients experienced Clavien–Dindo grade 1, while 12 had grade 2 and 4 had grade 3 complications. In the unsuccessful group, no complications were observed in 34 patients, while grade 1 complications were observed in 9 patients, grade 2 complications in 7 patients, and grade 3 complications in 3 patients. Complication rates were comparable between the two group (*p* = 0.729). Serum hemoglobin and creatinine levels were also similar between the groups preoperatively and postoperatively (*p* > 0.05 for all). Table 1 presents the demographic, clinical characteristics, and perioperative variables of the patients. A non-linear decreasing trend in stone-free rate was observed as the T.O.HO.-PCNL score of the patients increased (*p* = 0.244) (Table 2).

**Figure 2:**
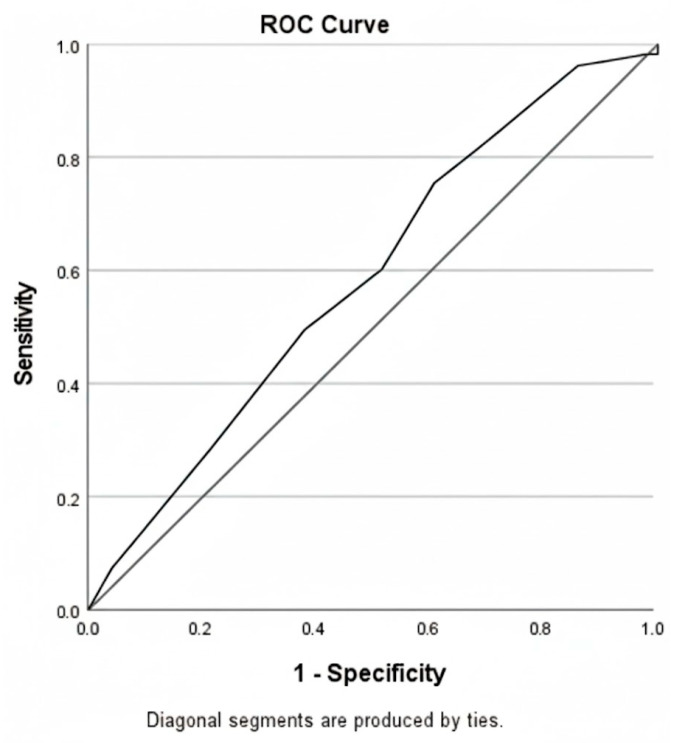
ROC curves for predicting success of PCNL based on T.O.HO.-PCNL score. AUC = 0.598, *p* = 0.046, 95% CI (0.506–0.690); Cut-off value = 8.5, sensitivity = 0.755, specificity = 0.412.

In the multivariate model, stone size emerged as an independent predictor of PCNL success. (OR: 1.076; 95% CI: 1.032–1.122; *p* < 0.001). Although the absence of hydronephrosis showed borderline statistical significance, it was not considered an independent predictor (OR: 2.006; 95% CI: 0.932–4.317; *p* = 0.075). The T.O.HO.-PCNL score was found to have no significant effect on success (OR: 1.031; 95% CI: 0.862–1.234; *p* = 0.735) (Table 3). Subsequently, ROC curves were generated to evaluate the effectiveness of the T.O.HO.-PCNL score in predicting success in PCNL. The area under the curve (AUC) of the T.O.HO.-PCNL score was 0.598, and when the cut-off value was set at 8.5, the sensitivity was 0.755 and the specificity was 0.412 (95% CI: 0.506–0.690; *p* = 0.046) (Figure 2).

## 4. Discussion

PCNL is widely accepted as the standard surgical intervention for large or complex stones. Although notable progress has been achieved in surgical techniques and instrumentation, serious complications can occur in some cases after PCNL, and complete stone-free survival may not be achieved. This highlights the need for more detailed evaluation of the clinical, anatomic, and stone-related factors that influence PCNL success. Furthermore, predictability of treatment outcomes is crucial for better patient counseling and planning the appropriate surgical approach [12,14]. Therefore, radiological and clinical scoring systems have been developed to stratify surgical risk and estimate success rates.

In a study comparing three different nomograms by Vicentini et al., it was shown that the GSS, STONE and CROES nomograms were similar in predicting PCNL success. (AUC: 0.653, AUC: 0.563, AUC: 0.641, respectively) [15]. In another study comparing the same three nomograms with 246 patients, the AUC values were 0.634, 0.671 and 0.670, respectively [16]. Bozkurt et al. showed that both the GSS and CROES nomograms showed comparable accuracy in predicting stone-free status after PCNL [17]. We also showed that the newly developed T.O.HO.-PCNL score was significant in predicting PCNL success. (AUC: 0.598, *p* = 0.046, 95% CI: 0.506–0.690). However, in multivariate regression analysis, the T.O.HO.-PCNL score alone was not significant as an independent predictor of PCNL failure. In this context, we believe it can only be helpful in clinical decision-making when evaluated in conjunction with anatomical and radiological parameters such as stone size. However, while similar AUC values have been obtained in other studies in the literature, we believe that AUC alone does not fully reflect the true clinical value of these scores. Indeed, nomograms are clinically meaningful not only for their statistical predictive power but also for their contributions to surgical planning, team coordination, and patient information [15]. Overall, our results suggest that the T.O.HO.-PCNL score alone may not be sufficient to predict PCNL success. In addition, the identified cut-off values did not provide adequate specificity. However, we believe that these limitations may be overcome through future prospective studies with larger patient populations, which may allow further refinement of the model and the development of a robust nomogram. Despite these shortcomings, due to its ease of application and standardization, the T.O.HO.-PCNL score may represent a practical nomogram that can be readily implemented in routine clinical practice in the future.

However, the primary purpose of the T.O.HO.-PCNL score is not to replace established predictors such as stone size, but rather to provide a structured assessment of overall case complexity. In this context, the score may assist preoperative operative planning, estimation of surgical difficulty, anticipation of prolonged operative time or the need for advanced strategies such as Endoscopic Combined Intrarenal Surgery (ECIRS), and optimization of team and resource allocation.

The differences in parameters used in nomograms sometimes lead to a lack of methodological standardization among scores. For example, Jeong et al. developed the Seoul National University Renal Stone Complexity (S-ReSC) score, which is independent of stone number and size and is associated with the complexity of stone distribution [18]. The AUC value for this score was reported as 0.860, which is significantly higher than the value obtained in our study. This score demonstrated high predictive power, particularly due to its consideration of the anatomic location and pelvicalyceal distribution of stones. This discrepancy may be explained by the fact that to the fact that the S-ReSC score assesses stone complexity based on anatomic distribution, while the T.O.HO.-PCNL score used in our study focuses more on stone size and operational variables. We also believe that the T.O.HO.-PCNL score is a simpler and more easily applicable scoring system compared with the S-ReSC score.

Hori et al. introduced the T.O.HO. scoring system in 2020 to estimate stone-free outcomes following RIRS [10]. Three main criteria are evaluated in this model: stone length (height) (1–5 points), stone location (occupied lesion) (1–3 points), and stone density (Hounsfield unit) (1–3 points). Based on these parameters, patients are assigned a total score between 3 and 11 points. Despite including a small number of variables, the original study reported a remarkably high predictive value (AUC: 0.83). Subsequent external validation studies also confirmed the predictive value of this scoring system (AUC: 0.799), and it was shown that the predictive accuracy increased further when stone volume was included in the model as an additional parameter (modified T.O.HO. (AUC: 0.821) [13].

To date, there is only one study in the literature in the context of using the T.O.HO. score for PCNL patients. Li et al. evaluated the original T.O.HO. score in patients undergoing supermini PCNL and standard PCNL and reported that this scoring system was a significant factor independently predicting stone-free rates [19]. However, this study applied the T.O.HO. system with its original RIRS-based parameters and does not directly reflect the anatomical difficulties or surgical complexity specific to PCNL. In this respect, the findings of Li et al. are important in demonstrating that the T.O.HO. score may have potential predictive power in different surgical approaches. However, considering the technical features of PCNL, the need to rearrange the system with parameters specific to this surgery becomes evident.

In the present study, we modified the T.O.HO. scoring system to classify the complexity of PCNL instead of its original application in RIRS. This modification is based on the understanding that the difficulty of PCNL is largely influenced by calyceal anatomy and stone distribution, and therefore presents significantly different dynamics than RIRS cases. Therefore, the “stone location” parameter was revised to more accurately reflect the anatomical difficulties of PCNL: renal pelvis (1 point), lower calyces (2 points), middle calyces (3 points), upper calyces (4 points), and stones located in multiple calyces (5 points). We believe that this revision allows the T.O.HO.-PCNL score to more accurately reflect the surgical difficulty inherent in PCNL and has the potential to assess operative outcomes with a procedure-specific, objective, and predictive approach.

In our study, we believe that one of the main reasons for the insufficient specificity of the T.O.HO.-PCNL score in predicting PCNL success is the inclusion of patients with complex staghorn stones. In clinical practice, these patients are known to be difficult to operate on and are frequently associated with higher complication rates. Due to the high likelihood of residual stones, surgical success rates are also lower in this subgroup. In recent years, robotic pyelolithotomy has emerged as an important alternative surgical option for such patients. In a recent study by Moretto et al., robotic pyelolithotomy was shown to be more successful than PCNL in patients with large renal stones [20]. We believe that excluding large staghorn stones in future studies may yield more objective results and contribute to further improvement of the T.O.HO.-PCNL score.

Mandal et al., in a prospective cohort of 248 PCNL patients, reported that complications of all Clavien-Dindo grades were significantly associated with higher GSS [21]. Similarly, another study by Singla et al. showed that GSS, STONE, and CROES scores were associated with the development of complications, although the correlation was weak [4]. Conversely, there are also results in the literature that contradict these findings. Noureldin et al. reported that the GSS and STONE scores and complication rates were not significantly correlated [22]. In this study, similarly, the T.O.HO.-PCNL score was found to have no significant predictive power in predicting the development of complications.

An idealized prediction model should combine the properties of accuracy, good calibration, simplicity, reproducibility, and clinical significance [18]. Moons et al. have clearly defined the methodological standards for conducting validation studies in the literature [23,24]. In our study, we adhered to these standards and carried out a highly methodologically accurate and reliable analysis process. The T.O.HO.-PCNL scoring system developed in this context aims to bring us one step closer to this ideal by reflecting the unique surgical dynamics of PCNL. All data in our study were obtained from computed tomography (CT) images. Because the CT findings, measurement, and evaluation criteria were standardized and objective for all physicians, this prevented any subjective interpretation from being included in the data collection process. In this respect, the score offers significant advantages in terms of both ease of application and high reproducibility. The score’s simple, practical, and clinically applicable structure suggests that it may serve as a valuable tool for predicting stone-free success and surgical difficulty. However, to achieve its goal of becoming an ideal prediction model, the T.O.HO.-PCNL score should be re-evaluated in different centers, with larger samples, and with a prospective design. Thus, both the external validity and calibration performance of the score can be revealed more clearly.

Accordingly, the present study should be interpreted primarily as an exploratory development and preliminary validation of a procedure-specific modification of the T.O.HO. scoring system for PCNL, rather than as a definitive predictive model. The primary objective was not to establish a standalone predictor of stone-free outcomes, but to investigate whether a simplified, anatomy-oriented scoring framework could reflect procedural complexity and support clinical decision-making in PCNL. In this context, the modest discriminative performance observed is consistent with an early-phase model development study and highlights the need for further refinement and validation.

There are several limitations affecting the results of our study. The most important of these is its retrospective design. Furthermore, despite the surgeons’ similar experience levels, minor differences in surgical techniques may have influenced the study results. Additionally, one potential shortcoming is that the analysis was limited to a single-center setting. Nevertheless, we consider that our validation of the T.O.HO.-PCNL score provides a novel contribution to the existing literature.

## 5. Conclusions

Our study involved the development of a modified T.O.HO.-PCNL score to reflect the anatomical challenges inherent in PCNL. The findings demonstrated significant performance in predicting PCNL success. The simplicity of the model and its reliance on objective parameters increase its clinical applicability. Furthermore, a higher T.O.HO.-PCNL score may help identify more complex cases that may require Endoscopic Combined Intrarenal Surgery (ECIRS) instead of standard PCNL in preoperative planning. However, our results did not provide sufficient specificity to define an optimal cut-off score. But, to achieve the goal of being an optimal prediction model, this score needs to be retested with prospective, multicenter, and external validation studies.

## Figures and Tables

**Table 1:** Comparison of demographic, clinical and stone-related parameters between successful and unsuccessful PCNL groups.

Variable	Total (*n* = 155)	Successful (*n* = 102)	Unsuccessful (*n* = 53)	*p*-Value
Sex (M/F)	102/53	64/38	38/15	0.265
Age (years)	49.99 ± 13.19	50.95 ± 13.59	48.15 ± 12.31	0.211
BMI (kg/m^2^)	28.86 ± 4.27	29.31 ± 4.25	27.91 ± 4.16	0.051
Stone side (Right/Left)	79/76	55/47	24/29	0.301
Stone location				0.723
–Multiple	66 (42.6%)	47 (46.0%)	19 (35.8%)	
–Renal pelvis	63 (40.6%)	35 (34.3%)	28 (52.8%)	
–Lower pole	20 (12.9%)	15 (14.7%)	5 (9.4%)	
–Middle pole	5 (3.2%)	4 (3.9%)	1 (1.9%)	
–Upper pole	1 (0.6%)	1 (1.0%)	0 (0.0%)	
Stone size (mm)	30.40 ± 10.95	27.45 ± 8.71	35.30 ± 11.50	<0.01
Stone density (HU)	1029.47 ± 352.55	990.75 ± 351.83	1090.38 ± 347.12	0.095
Preoperative hydronephrosis (%)	93 (60.0%)	54 (52.9%)	39 (73.6%)	<0.05
Residual stone size (mm)	2.06 ± 2.41	0.51 ± 1.12	5.96 ± 0.66	<0.01
Operation time (min)	119.90 ± 45.03	110.74 ± 43.19	134.89 ± 45.75	<0.01
Hospital stay (days)	4.29 ± 2.09	3.75 ± 1.70	5.26 ± 2.34	<0.01
Complications (None/G1/G2/G3)	95/34/19/7	61/25/12/4	34/9/7/3	0.729
Preoperative Hb (g/dL)	14.38 ± 1.71	14.40 ± 1.72	14.34 ± 1.71	0.824
Postoperative Hb (g/dL)	12.73 ± 2.03	12.83 ± 2.25	12.56 ± 1.70	0.451
Preoperative creatinine (mg/dL)	1.13 ± 1.40	1.09 ± 1.21	1.18 ± 1.62	0.715
Postoperative creatinine (mg/dL)	0.99 ± 0.41	0.99 ± 0.44	0.98 ± 0.37	0.891
T.O.HO.–PCNL score	9.49 ± 2.23	9.21 ± 2.27	9.96 ± 2.10	<0.05

**Table 2:** Success rate after PCNL according to the T.O.HO.-PCNL score.

T.O.HO.-PCNL Score	Succes Rate, % (*n*)
4	0.0% (0/1)
5	100% (2/2)
6	93.3% (14/15)
7	69.6% (16/23)
8	71.4% (10/14)
9	52.9% (9/17)
10	70.0% (14/20)
11	59.3% (16/27)
12	60.7% (17/28)
13	50.0% (4/8)
Total	65.8% (102/155) *p* = 0.244 (Chi-square test)

**Table 3:** Multivariate logistic regression model for independent predictors of PCNL success.

Parameter	OR (95% CI)	*p*
Stone size	1.076 (1.032–1.122)	<0.001
Absence of hydronephrosis	2.006 (0.932–4.317)	0.075
Complications	1.042 (0.813–1.182)	0.875
T.O.HO.-PCNL score	1.031 (0.862–1.234)	0.735

## Data Availability

The original contributions presented in the study are included in the article, further inquiries can be directed to the corresponding authors.

## References

[1] Wu Y., Gong J., Qiu C., Yin G., Yuan P. (2025). Nomogram for Predicting Postoperative Major Bleeding in Percutaneous Nephrolithotomy: A Retrospective Study. Eur. Urol. Open Sci..

[2] Wang H.-H., Lin K.-J., Chu S.-H., Chen H.-W., Chiang Y.-J., Lin P.-H., Wang T.-M., Liu K.-L. (2014). The impact of climate factors on the prevalence of urolithiasis in Northern Taiwan. Biomed. J..

[3] Türk C., Petřík A., Sarica K., Seitz C., Skolarikos A., Straub M. (2016). EAU Guidelines on Diagnosis and Conservative Management of Urolithiasis. Eur. Urol..

[4] Singla A., Khattar N., Nayyar R., Mehra S., Goel H., Sood R. (2017). How practical is the application of percutaneous nephrolithotomy scoring systems? Prospective study comparing Guy’s Stone Score, S.T.O.N.E. score and the Clinical Research Office of the Endourological Society (CROES) nomogram. Arab. J. Urol..

[5] Vernez S.L., Okhunov Z., Motamedinia P., Bird V., Okeke Z., Smith A. (2016). Nephrolithometric Scoring Systems to Predict Outcomes of Percutaneous Nephrolithotomy. Rev. Urol..

[6] Thomas K., Smith N.C., Hegarty N., Glass J.M. (2011). The Guy’s stone score—Grading the complexity of percutaneous nephrolithotomy procedures. Urology.

[7] Mishra S., Sabnis R.B., Desai M. (2012). Staghorn morphometry: A new tool for clinical classification and prediction model for percutaneous nephrolithotomy monotherapy. J. Endourol..

[8] Okhunov Z., Friedlander J.I., George A.K., Duty B.D., Moreira D.M., Srinivasan A.K., Hilelsohn J., Smith A.D., Okeke Z. (2013). S.T.O.N.E. nephrolithometry: Novel surgical classification system for kidney calculi. Urology.

[9] Smith A., Averch T.D., Shahrour K., Opondo D., Daels F.P., Labate G., Turna B., de la Rosette J.J., CROES PCNL Study Group (2013). A nephrolithometric nomogram to predict treatment success of percutaneous nephrolithotomy. J. Urol..

[10] Hori S., Otsuki H., Fujio K., Kobayashi H., Nagao K., Nakajima K., Mitsui Y. (2020). Novel prediction scoring system for simple assessment of stone-free status after flexible ureteroscopy lithotripsy: T.O.HO. score. Int. J. Urol..

[11] Bulut S., Yahsi S., Ceviz K., Esengen S., Gültekin H. (2024). External validation of Ito’s nomogram and T.O.HO. scoring system in flexible ureterorenoscopy. BMC Urol..

[12] Senel S., Kasap Y., Kizilkan Y., Tastemur S., Ozden C. (2022). External validation of the T.O.HO. score as predictor of success after retrograde intrarenal surgery. BMC Urol..

[13] Polat S., Danacioglu Y., Yarimoglu S., Soytas M., Erdogan A., Teke K., Degirmenci T., Tasci A. (2023). External validation of the current scoring systems and development of a novel scoring system to predict stone-free rates after retrograde intrarenal surgery in patients with cumulative stone diameter of 2–4 cm. Actas Urol. Esp..

[14] Sfoungaristos S., Gofrit O.N., Mykoniatis I., Landau E.H., Katafigiotis I., Pode D., Constantinides C.A., Duvdevani M. (2016). External validation of Resorlu-Unsal stone score as predictor of outcomes after retrograde intrarenal surgery. Int. Urol. Nephrol..

[15] Vicentini F.C., Serzedello F.R., Thomas K., Marchini G.S., Torricelli F.C.M., Srougi M., Mazzucchi E. (2017). What is the quickest scoring system to predict percutaneous nephrolithotomy outcomes? A comparative study among S.T.O.N.E score, guy’s stone score and croes nomogram. Int. Braz. J. Urol..

[16] Labadie K., Okhunov Z., Akhavein A., Moreira D.M., Moreno-Palacios J., del Junco M., Okeke Z., Bird V., Smith A.D., Landman J. (2015). Evaluation and comparison of urolithiasis scoring systems used in percutaneous kidney stone surgery. J. Urol..

[17] Bozkurt I.H., Aydogdu O., Yonguc T., Yarimoglu S., Sen V., Gunlusoy B., Degirmenci T. (2015). Comparison of Guy and Clinical Research Office of the Endourological Society Nephrolithometry Scoring Systems for Predicting Stone-Free Status and Complication Rates After Percutaneous Nephrolithotomy: A Single Center Study with 437 Cases. J. Endourol..

[18] Jeong C.W., Jung J.W., Cha W.H., Lee B.K., Lee S., Jeong S.J., Hong S.K., Byun S.S., Lee S.E. (2013). Seoul National University Renal Stone Complexity Score for Predicting Stone-Free Rate after Percutaneous Nephrolithotomy. PLoS ONE.

[19] Li Y., Pan L. (2025). Analysis of factors affecting the efficacy and stone clearance rate of super-mini PCNL (SMP) versus standard PCNL (sPCNL) in the treatment of different sizes of renal stones. Int. Urol. Nephrol..

[20] Moretto S., Zazzara M., Marino F., Ragonese M., Scarcia M., Gradilone U., Russo P., Montesi M., Lentini N., Pastorino R. (2024). Percutaneous nephrolithotomy vs. robotic pyelolithotomy for large renal stones: An inverse probability treatment weighting analysis. Minerva Urol. Nephrol..

[21] Mandal S., Goel A., Kathpalia R., Sankhwar S., Singh V., Sinha R.J. (2012). Prospective evaluation of complications using the modified Clavien grading system, and of success rates of percutaneous nephrolithotomy using Guy’s Stone Score: A single-center experience. Indian. J. Urol..

[22] Noureldin Y.A., Elkoushy M.A., Andonian S. (2015). Which is better? Guy’s versus S.T.O.N.E. nephrolithometry scoring systems in predicting stone-free status post-percutaneous nephrolithotomy. World J. Urol..

[23] Moons K.G.M., Kengne A.P., E Grobbee D., Royston P., Vergouwe Y., Altman D.G., Woodward M. (2012). Risk prediction models: II. External validation, model updating, and impact assessment. Heart.

[24] Moons K.G.M., Kengne A.P., Woodward M., Royston P., Vergouwe Y., Altman D.G., E Grobbee D. (2012). Risk prediction models: I. Development, internal validation, and assessing the incremental value of a new (bio)marker. Heart.

